# Digital Ischemia in an Extreme Preterm Infant Treated with Nitroglycerin Patch

**DOI:** 10.1155/2024/2255756

**Published:** 2024-02-28

**Authors:** Mansour Al Qurashi, Abdulaziz Al-Khotani, Farzeen Mohtisham, Eman AlRaddadi, Heba AlShaikh, Alqassem Y. Hakami, Syed Sameer Aga

**Affiliations:** ^1^Department of Pediatrics, Ministry of National Guard Health Affairs (MNGHA), King Saud Bin Abdul Aziz University for Health Sciences (KSAU-HS), WR King Abdulaziz Medical City, Jeddah, Saudi Arabia; ^2^King Abdullah International Medical Research Centre (KAIMRC), Ministry of National Guard Health Affairs (MNGHA), King Abdulaziz Medical City, Jeddah, Saudi Arabia; ^3^Department of Pediatrics, Umm AlQura University, Makkah, Saudi Arabia; ^4^Department of Pediatrics, Dr. Soliman Fakeeh Hospital, Jeddah, Saudi Arabia; ^5^Department of Basic Sciences, College of Science and Health Professions, King Abdulaziz Medical City, Jeddah, Saudi Arabia; ^6^Department of Basic Medical Sciences, College of Medicine, King Saud Bin Abdul Aziz University for Health Sciences (KSAU-HS), King Abdulaziz Medical City, Jeddah, Saudi Arabia

## Abstract

Ischemic limb lesions occasionally occur in neonates admitted to neonatal intensive care units. Known risk factors include the placement of arterial catheters, arterial punctures to obtain blood samples, and the use of vasoactive/vasopressor medications for hypotension. Prolonged peripheral tissue ischemia may result in serious complications, and successful management depends on early detection, proper assessment, and the institution of appropriate intervention. Currently, there is no standard approach for the management of peripheral tissue ischemia in extreme preterm infants. Topical nitroglycerine use is one of the promising options used to manage ischemic limb injuries in neonates, as demonstrated in several case reports. We report a case of digital ischemia in an extreme preterm infant with no clear risk factors except extreme prematurity, which recovered after topical nitroglycerine therapy.

## 1. Background/Introduction

Neonatal peripheral tissue ischemia following localized thrombosis is one of the most serious complications of vascular catheterization. The causes may vary depending on the thromboembolic event, vasospasm, or medication extravasation, resulting in tissue ischemia [1, 2]. If not treated timely, prolonged peripheral tissue ischemia may lead to serious complications such as tissue necrosis, irreversible loss of function, and gangrene of the affected extremity [3]. Successful management depends on early recognition, effective assessment, and an appropriate choice of therapy.

The current approach for the management of peripheral tissue ischemia typically involves conservative steps, anticoagulation therapy, and/or surgical intervention; however, pharmaco-surgical options in preterm infants are of limited use due to the high risk of complications like intraventricular hemorrhage (IVH) [4] and the tiny body size, which make the role of surgical intervention, difficult if not impossible. Nitroglycerin is a vasodilator commonly used in adults to manage angina and acute coronary syndrome. To date, evidence supporting the use of topical nitroglycerin for the management of neonatal tissue ischemia of various etiologies has been limited to a several published case reports [4]. This case report demonstrates the successful use of a nitroglycerin patch in an extremely preterm infant to treat severe peripheral tissue ischemia.

## 2. Case Summary

**Figure 1:**
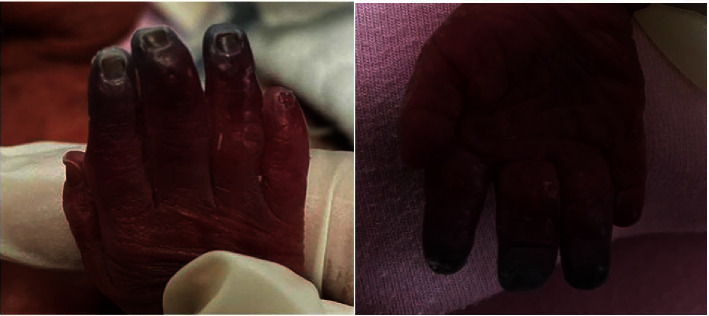
Significant deep blush discoloration of index, middle and ring fingers few hours from the onset from the event.

A baby girl was born at 24 weeks gestation with a weight of 703 grams and had experienced a range of expected prematurity sequelae that included respiratory distress syndrome and grade 2 intraventricular hemorrhage (IVH). She had an umbilical venous catheter inserted at birth for venous access and to provide total parenteral nutrition but no umbilical arterial catheter nor arterial cannulation were attempted, particularly in the affected arm/hand. At the age of 6 days, she developed gradual mild bluish discoloration affecting mainly the second, third, and fourth fingers of her right hand, and despite initial management with warm compression and other conservative steps, the discoloration persisted and even worsened (Figure 1). There were no attempts at peripheral venous cannulation in the affected arm or hand.

The urgent Doppler study revealed a mild reduction in blood flow in the radial and ulnar arteries of the affected hand, although it remained patent. Therapeutic anticoagulation was considered in consultation with the hematology service but ultimately not started due to the presence of a recent intraventricular hemorrhage (IVH) and the absence of a clear thrombus. The managing team initiated treatment using a nitroglycerin patch placed over affected hand fingers. 4 cm^2^ nitroglycerin patch was used, as every 1 cm^2^ of application delivers approximately 0.026 mg of nitroglycerine per hour. The 4 cm^2^ strip was replaced every 12 hours for the next 5 days. The baby was monitored for the known side effects that include methemoglobinemia subsequent to the use of the GTN patch and hypotension, which luckily were not observed during the 5 -day treatment.

**Figure 2:**
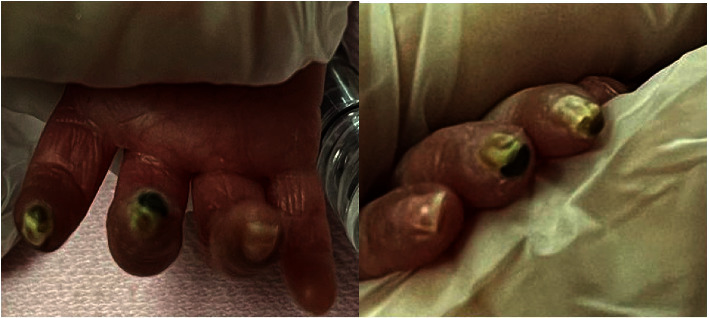
Residual distal ischemic changes of the affected index and middle fingers with complete recovery of the ring fingers.

**Figure 3:**
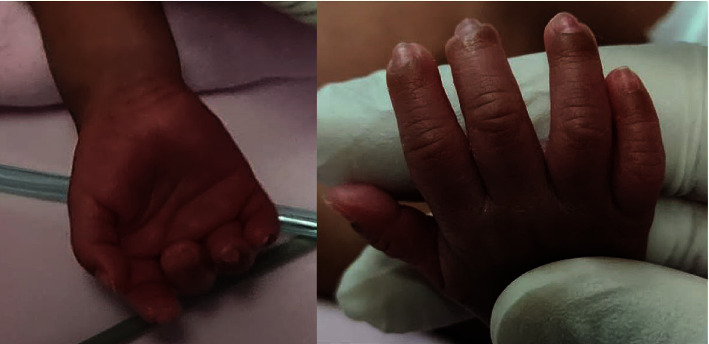
Marked resolution of the ischemic changes of affected fingers 4 weeks later.

Within a few hours after the application of the topical nitroglycerine patch, a noticeable gradual improvement was observed until the ischemic changes markedly improved over the next 5–7 days (Figure 2). The affected hand fingers ischemic changes continued to improve until complete resolution 4 weeks later (Figure 3).

## 3. Discussion

Ischemic skin lesions of the extremities in neonates are exceptionally rare. They are usually associated with catheterization procedures in neonates with other risk factors like the use of inotropes, hypercoagulable state, and sepsis [5]. It has been reported in association with episodes of severe hypoxia and hypoperfusion of the distal extremity, especially in sick neonates [6]. In newborn infants, the incidence of permanent ischemic damage to digits or limbs following arterial catheterizations is reported to be up to 1.6%; however, the incidence of transient ischemia has been reported to be around 6%, and following umbilical arterial catheterization is reported to be around 1%.

Limited data is available on the therapeutic approach to persistent ischemic incidents in extreme preterm infants, complicating the use of peripheral and central arterial catheters. Typically, these events are managed by immediate removal of the catheters and conservative management including elevation and warming of the limb, which usually lead to spontaneous resolution in the majority of cases. Systemic anticoagulation might be useful in persistent ischemic events especially if the underlying pathology is thrombosis but it has its risks in extreme preterm infants who are at high risk of intraventricular hemorrhage and hence is not always feasible in neonates [7]. Severe ischemia can lead to gangrene and may necessitate surgical debridement and loss of the digit or the extremity [8]. Topical nitroglycerin as either cream or a patch has been used successfully to treat digital ischemia in several reported cases [9]. The primary mechanism of action of nitroglycerin is relaxation of vascular smooth muscles, leading to dilation of both arterial and venous beds. The vasodilation effect of nitroglycerin is attributed to increased levels of nitric oxide, which activates the enzyme guanylyl cyclase, leading to an increase in cellular nucleotide cyclic guanosine monophosphate (cGMP), and subsequently, cGMP results in dephosphorylation of myosin in smooth muscle and relaxation [10].

The use of a 2% nitroglycerin ointment was previously reported to be successful in the management of neonatal tissue ischemia caused by dopamine extravasation [10, 11] or as a complication of peripheral arterial line placement [11–15]. On the other hand, successful use of a nitroglycerin patch for the treatment of tissue injury caused by total parental nutrition extravasation, umbilical arterial line, or peripheral line is limited to sporadic case reports [16–19]. It is important to note that there is consistency among all previously published reports in the improvement of blood perfusion to the affected extremity and complete recovery in newborns following topical nitroglycerin therapy. In this case, nitroglycerin treatment was started 3 hours after the appearance of symptoms, and there was gradual improvement on the following 5 days of therapy until significant recovery was achieved after 4 weeks. The pharmacokinetic profile of transdermal nitroglycerin in infants is still not known; however, in adults, topical nitroglycerin is well absorbed topically through intact skin, and the systemic bioavailability of the nitroglycerin patch could reach 90%, with an onset of action of approximately 30–60 minutes and a half-life of 1–3 minutes [20]. Further research and studies are needed to support the efficacy and safety of nitroglycerin patches as a noninvasive and relatively safe therapeutic option for neonatal ischemic events. In adults, common side effects of nitroglycerin treatment include headache, tachycardia, and hypotension; however, limited data suggest these side effects are rare in the pediatric and neonatal population [21].

While quite promising, further studies are needed to support the efficacy and safety of nitroglycerin patches or topical cream as a noninvasive and relatively safe therapeutic option for neonatal ischemic events.

However, we do acknowledge that the use of nitroglycerin patches has its own known risk factors as highlighted by this case as follows: (a) Arterial nor central lines were there, except the umbilical venous line, which is anatomically away from the ischemic hand. Furthermore, the baby was hemodynamically stable with normal BP for his age, and thirdly, the blood culture was negative for any sepsis markers, which makes the link to sepsis unlikely. Moreover, extremely low gestational age, an immature hemostatic system, vulnerable and narrow blood vessel diameter, and overall being at higher risk for adverse outcomes including ischemic complications such as tissue necrosis and gangrene [4, 10, 14, 22].

## 4. Conclusion

This case report demonstrates a successful treatment of hand ischemia in extreme low-birth-weight preterm infants using a nitroglycerin patch for 5 days. As with any treatment modality, the risks and benefits must be assessed on a case-by-case basis, and the managing team must consider the individual case settings and potential side effects before initiating treatment with nitroglycerin patches in neonates. Nonetheless, this case report provides a promising story about how the nitroglycerine patch may be a viable option for treating ischemic events in extreme preterm weight infants.

## Data Availability

Data used in this study are made available upon request.
